# Concordance Between Multiplex-PCR Resistance-Gene Detection and MIC-Based Susceptibility in Gram-Negative Clinical Isolates from a Romanian Tertiary Hospital

**DOI:** 10.3390/antibiotics15101027

**Published:** 2026-10-15

**Authors:** Alexandra Bucătaru, Andrei Osman, Ovidiu Mircea Zlatian, Eugen Mihail Arnautu, Andrei Theodor Bălășoiu, Maria Bălășoiu, Georgiana Cristiana Camen, Alex Ioan Salan, Andreea-Loredana Golli, Alice Elena Ghenea

**Affiliations:** 1 Medical Doctoral School, University of Medicine and Pharmacy of Craiova, 200349 Craiova, Romania; eugen.arnautu@umfcv.ro; 2 Department of Anatomy and Embryology, University of Medicine and Pharmacy of Craiova, 200349 Craiova, Romania; andrei.osman@umfcv.ro; 3 Otorhinolaringology Department, Emergency County Hospital of Craiova, 200642 Craiova, Romania; 4 Department of Microbiology, University of Medicine and Pharmacy of Craiova, 200349 Craiova, Romania; ovidiu.zlatian@umfcv.ro (O.M.Z.); maria.balasoiu@umfcv.ro (M.B.); alice.ghenea@umfcv.ro (A.E.G.); 5 Clinical Laboratory, Emergency County Hospital of Craiova, 200642 Craiova, Romania; 6 Department of Ophthalmology, University of Medicine and Pharmacy of Craiova, 200349 Craiova, Romania; andrei_theo@yahoo.com; 7 Center for Studies in Morphological Microscopy and Immunology, University of Medicine and Pharmacy of Craiova, 200349 Craiova, Romania; 8 Radiology and Medical Imaging Department, University of Medicine and Pharmacy of Craiova, 200349 Craiova, Romania; 9 Department of Oral and Maxillofacial Surgery, University of Medicine and Pharmacy of Craiova, 200349 Craiova, Romania; alex.salan@umfcv.ro; 10 Department of Public Health and Management, University of Medicine and Pharmacy of Craiova, 200349 Craiova, Romania; andreea.golli@umfcv.ro

**Keywords:** carbapenemase-producing Enterobacterales, phenotype-genotype correlation, Unyvero multiplex-PCR, VITEK^®^2, KPC, OXA-48, antibiotic stewardship, multidrug resistance

## Abstract

**Background/Objectives:** Rapid molecular detection of antimicrobial resistance determinants has the potential to optimize antimicrobial therapy before conventional antimicrobial susceptibility testing becomes available. However, the clinical utility of these assays depends on the concordance between detected resistance genes and phenotypic susceptibility. This study aimed to evaluate the genotype–phenotype concordance of carbapenemase and β-lactamase genes detected by the Unyvero multiplex-PCR platform with minimum inhibitory concentration (MIC)-based susceptibility profiles obtained using the phenotypic AST comparator method in Gram-negative clinical isolates. **Methods:** A total of 117 non-duplicate Gram-negative clinical isolates recovered from hospitalized patients at a Romanian tertiary-care hospital were analyzed. Resistance determinants, including *bla*KPC, *bla*OXA-48, *bla*OXA-23, *bla*CTX-M, *bla*TEM, together with aminoglycoside, fluoroquinolone, and sulfonamide resistance markers, were identified using the Unyvero multiplex-PCR system. Phenotypic antimicrobial susceptibility testing was performed using the VITEK^®^2 automated system according to EUCAST criteria. Genotype–phenotype concordance was evaluated using sensitivity, specificity, positive predictive value (PPV), negative predictive value (NPV), and Cohen’s κ coefficient. **Results:** Overall, 78.8% (78/99) of isolates were classified as non-susceptible. *bla*OXA-48 was the most frequently detected carbapenemase gene (n = 12), followed by *bla*KPC (n = 9) and *bla*OXA-23 (n = 3). *bla*KPC demonstrated a sensitivity of 100% for predicting imipenem resistance in Enterobacterales, with moderate agreement (κ = 0.58), whereas *bla*OXA-23 showed complete diagnostic performance (100% sensitivity and PPV) for carbapenem resistance in *Acinetobacter baumannii*. In contrast, all *bla*OXA-48-positive Enterobacterales remained phenotypically susceptible to carbapenems, indicating poor genotype–phenotype concordance. Likewise, *bla*CTX-M, *gyr*A83/87, and *qnr*A/B/S showed limited diagnostic performance for corresponding phenotypic resistance. **Conclusions**: Phenotype–genotype concordance varies across resistance determinants and Gram-negative bacterial species. Detection of *bla*KPC and *bla*OXA-23 may support early optimization of antimicrobial therapy, whereas *bla*OXA-48 should be interpreted together with conventional phenotypic antimicrobial susceptibility testing. Integrating rapid molecular diagnostics with phenotypic susceptibility testing may improve antimicrobial stewardship and optimize the management of multidrug-resistant Gram-negative infections.

## 1. Introduction

Carbapenem-resistant Enterobacterales (CRE) have established themselves as a major threat to global public health. The World Health Organization (WHO) has classified carbapenem-resistant Gram-negative bacteria as a critical priority pathogen for which new therapeutic strategies are urgently needed [1,2]. Infections caused by CRE are associated with limited treatment options, increased morbidity and mortality, prolonged hospitalization, and substantial healthcare costs [3,4]. The rapid global dissemination of these organisms has been driven by extensive use of broad-spectrum antibiotics, the expansion of high-risk bacterial clones, and the horizontal transfer of resistance determinants carried by mobile genetic elements [3,5,6]. Consequently, carbapenem-resistant Gram-negative pathogens have become a major focus of antimicrobial stewardship and infection prevention programs worldwide.

In addition to Enterobacterales, other Gram-negative pathogens, particularly non-fermenting bacteria such as *Pseudomonas aeruginosa* and *Acinetobacter baumannii*, represent major therapeutic and epidemiological challenges within the hospital healthcare setting. These organisms are frequently implicated in severe healthcare-associated infections, including ventilator-associated pneumonia, bloodstream infections, and complicated urinary tract infections [7]. Their remarkable ability to survive in hospital environments and accumulate multiple resistance determinants contributes to the emergence of multidrug-resistant phenotypes and further complicates antimicrobial therapy [7,8].

Carbapenem resistance in Enterobacterales is most commonly mediated by the production of carbapenemases, a diverse group of β-lactamases capable of hydrolyzing carbapenems and other β-lactam antibiotics. The most clinically relevant carbapenemases belong to Ambler classes A, B, and D, including serine carbapenemases such as *Klebsiella pneumoniae* carbapenemase (KPC), metallo-β-lactamases such as New Delhi metallo-β-lactamase (NDM), and oxacillinases such as OXA-48–like enzymes [5,6]. These enzymes differ in geographic distribution, substrate specificity, and susceptibility to β-lactamase inhibitors, leading to considerable variability in therapeutic options. Phenotypic cephalosporin resistance in isolates without detectable *bla*CTX-M may reflect mechanisms not targeted by the panel, but these were not investigated here. Several are plausible in this cohort. *E. cloacae*, *C. freundii* and *S. marcescens* carry chromosomal inducible ampC, and derepressed mutants confer resistance to third-generation cephalosporins, further complicating the resistance patterns observed in clinical isolates [6,9]. These explanations rest on the published literature and were not tested in this study. Establishing them would require phenotypic confirmatory testing, expression analysis, or whole-genome sequencing.

Historically, treatment options for CRE infections have relied on a limited number of last-line antimicrobial agents, including polymyxins, tigecycline, aminoglycosides, and fosfomycin [3,10]. However, the clinical utility of these agents is often limited by suboptimal pharmacokinetics, variable therapeutic efficacy, and significant toxicity. For instance, polymyxins remain associated with considerable nephrotoxicity, whereas tigecycline achieves low serum concentrations, limiting its use in bloodstream infections [10]. As a result, infections caused by carbapenem-resistant organisms are frequently associated with poor clinical outcomes and high mortality rates, particularly among critical care patients [4,11].

In recent years, the development of novel β-lactam/β-lactamase inhibitor (BL/BLI) combinations has expanded the therapeutic options available for infections caused by carbapenem-resistant organisms. Agents such as ceftazidime–avibactam and meropenem–vaborbactam have demonstrated improved activity against specific carbapenemase-producing strains and have been associated with favorable clinical outcomes in observational studies and systematic reviews [11,12]. An important aspect of the clinical effectiveness of these agents is the underlying resistance mechanism, underscoring the need for rapid identification of resistance determinants to guide antimicrobial therapy.

Traditional antimicrobial susceptibility testing remains the cornerstone of antimicrobial therapy selection; however, these methods typically require 24–48 h to provide adequate results. In contrast, rapid molecular diagnostic technologies can detect bacterial pathogens and resistance genes within a significantly shorter timeframe, potentially enabling earlier optimization of antimicrobial therapy [13]. Syndromic multiplex PCR systems, such as the Unyvero platform, allow simultaneous detection of multiple bacterial pathogens and resistance determinants directly from clinical samples [14,15]. Nevertheless, the detection of resistance genes does not always correlate with phenotypic antimicrobial resistance, and differences between genotypic and phenotypic bacterial profiles may complicate the clinical interpretation of molecular findings [16].

Despite the growing global burden of carbapenem-resistant Gram-negative bacteria, data regarding the relationship between molecular resistance markers and phenotypic susceptibility patterns remain limited in many regions, particularly in Eastern Europe. Understanding the local distribution of resistance mechanisms and their correlation with antimicrobial susceptibility profiles is essential for improving therapeutic decision-making and antimicrobial stewardship strategies at institutional levels.

Therefore, the objective of the present study is to evaluate the relationship between molecular detection of resistance genes and phenotypic antimicrobial susceptibility patterns among Gram-negative clinical isolates recovered from a tertiary care hospital in Craiova, Romania. Specifically, we analyzed the distribution of resistance genes detected by multiplex PCR and assessed their concordance with MIC-based susceptibility results obtained through automated antimicrobial susceptibility testing systems. By integrating molecular and phenotypic data, this study aims to provide clinically relevant insights into the interpretation of rapid diagnostic results and their potential role in guiding antimicrobial therapy for infections caused by carbapenem-resistant Gram-negative bacteria.

## 2. Results

A total of 117 samples were analyzed. Among these were 23 blood cultures, 10 ear swabs, 10 nasopharyngeal swabs, 7 sinus aspirates, 8 sputum specimens, 7 throat swabs, 18 tracheal aspirates, 16 urines, and 18 wound swabs.

**Figure 1:**
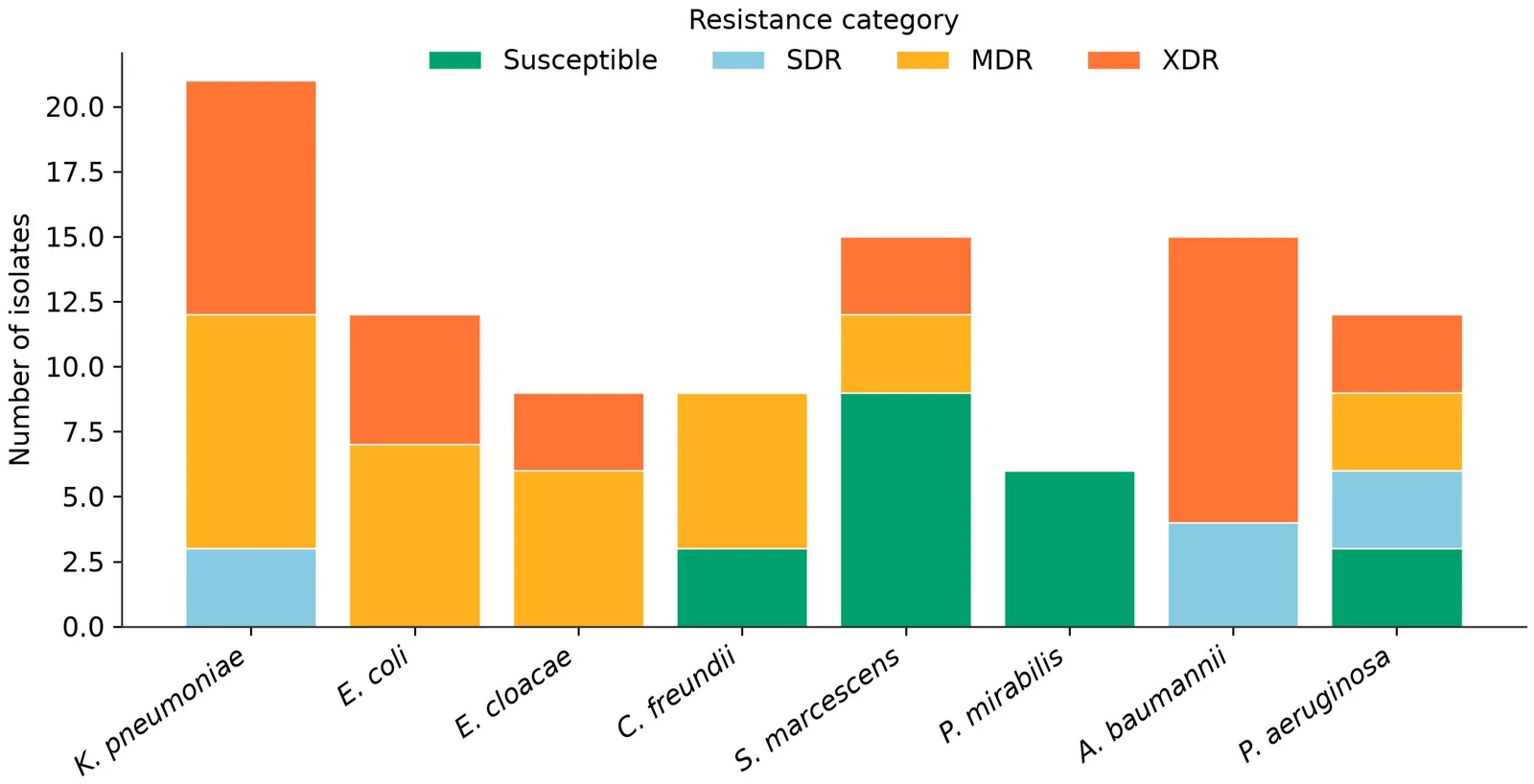
Distribution of Gram-negative isolates by species and resistance category.

A total of 117 isolates underwent molecular characterization. After exclusion of 18 *Stenotrophomonas maltophilia* isolates because of intrinsic carbapenem resistance, 99 isolates were retained for the primary resistance-category and genotype–phenotype analyses, which encompassed eight Gram-negative species (Table 1 and Figure 1). Overall, 78/99 (78.8%) isolates were classified as non-susceptible. Within the Gram-negative species tested, *Escherichia coli* (12/12, 100.0%), *Klebsiella pneumoniae* (21/21, 100.0%), *Enterobacter cloacae* (9/9, 100%) and *Acinetobacter baumannii* (15/15, 100.0%) showed highest non-susceptible status, followed by *Pseudomonas aeruginosa* (9/12, 75.0%), *Citrobacter freundii* (6/9, 66.7%), *Serratia marcescens* (6/15, 40.0%), and *Proteus mirabilis* (0/6, 0%).

Thirty-four strains were classified as MDR including 9 *Klebsiella pneumoniae*, 7 *Escherichia coli*, and 3 *Pseudomonas aeruginosa*, highlighting the severity of resistance in these strains.

### 2.1. Distribution of Resistance Genes

A total of 39 β-lactamase and carbapenemase gene detections were recorded across the 117 Gram-negative isolates (Table 2). The most frequently detected carbapenemase gene was *bla*OXA-48 (n = 12), identified in *Klebsiella pneumoniae*, *Escherichia coli*, *Citrobacter freundii*, and *Serratia marcescens*. *bla*KPC was detected in nine isolates: three *K. pneumoniae*, three *E. coli*, and three *Pseudomonas aeruginosa*. *bla*OXA-23 was detected in three *Acinetobacter baumannii* isolates. Among the other β-lactamase genes, *bla*CTX-M was detected in nine Enterobacterales isolates and *bla*TEM in six, whereas *bla*SHV was not detected.

**Figure 2:**
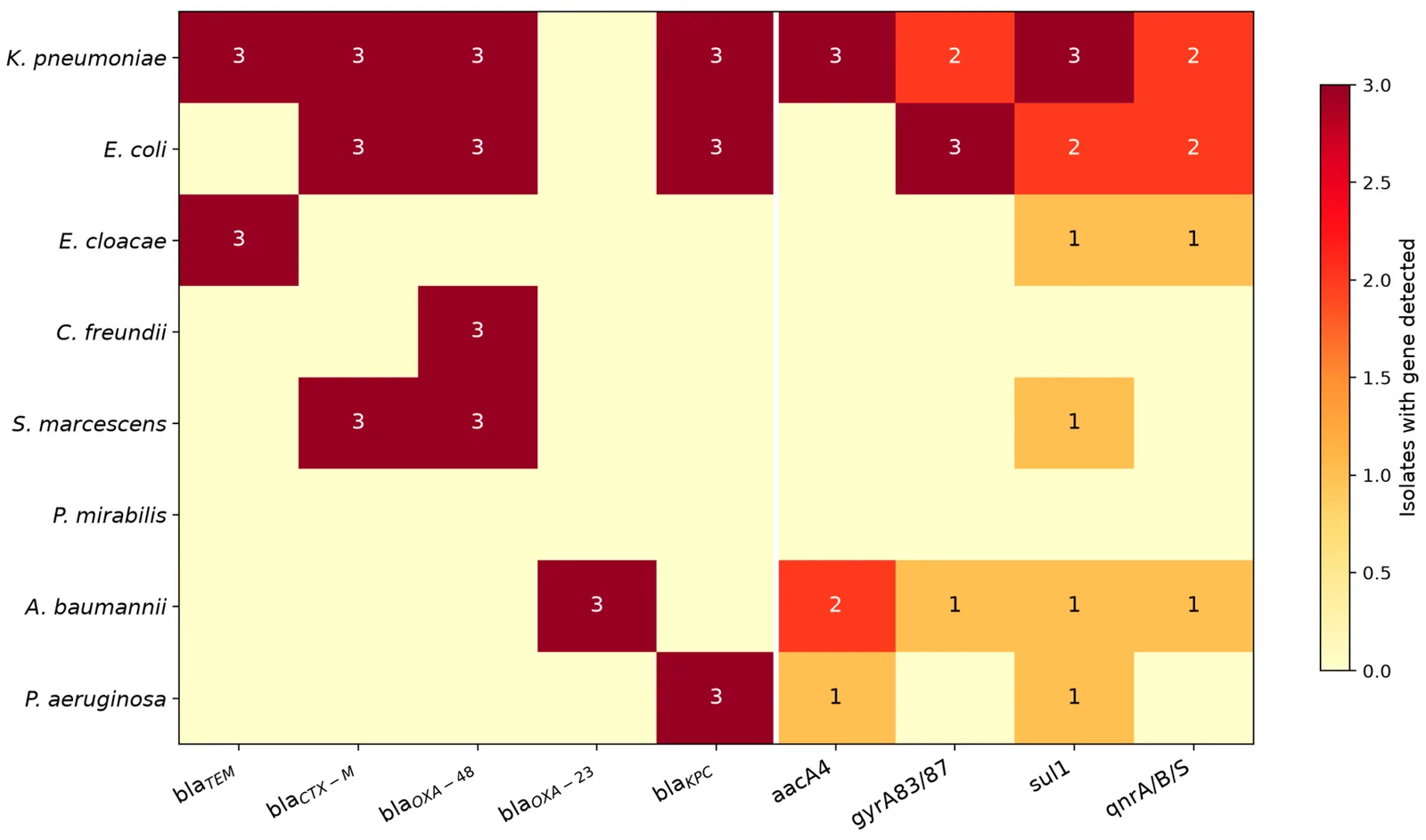
Species-level distribution of resistance genes detected by Unyvero multiplex PCR among the 99 isolates included in the primary analysis. Cell values represent the number of isolates of each species in which the corresponding marker was detected. Blank cells indicate no detection. The first five columns represent β-lactamase and carbapenemase genes; the remaining columns represent aminoglycoside, fluoroquinolone, and sulfonamide resistance markers.

Among the 99 isolates included in the primary analysis, aacA4 and *gyr*A83/87 were each detected in six isolates, *qnr*A/B/S in six, and *sul*1 in nine. The species-level distribution of all detected resistance markers is shown in Figure 2. No investigated resistance marker was detected in *Proteus mirabilis*.

### 2.2. Phenotype–Genotype Concordance

#### 2.2.1. Carbapenemase Genes (KPC and OXA-48)

**Figure 3:**
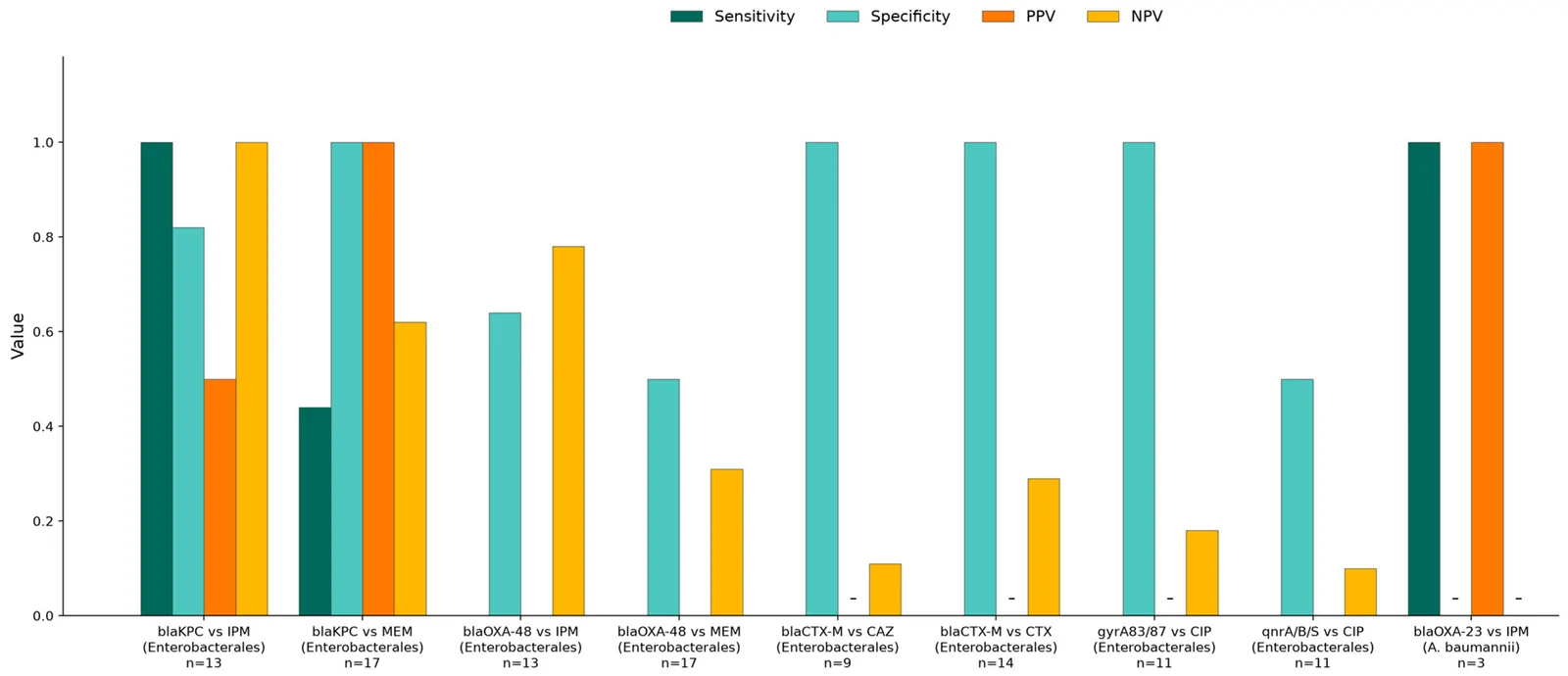
Diagnostic performance of selected molecular resistance markers against MIC-based phenotypic resistance. Bars represent sensitivity, specificity, positive predictive value (PPV), and negative predictive value (NPV). Measures that could not be calculated because of a zero denominator are not shown. Denominators represent isolates with paired molecular and interpretable phenotypic results and therefore differ between gene–antimicrobial comparisons. CAZ, ceftazidime; CTX, cefotaxime; CIP, ciprofloxacin; IPM, imipenem; MEM, meropenem.

**Figure 4:**
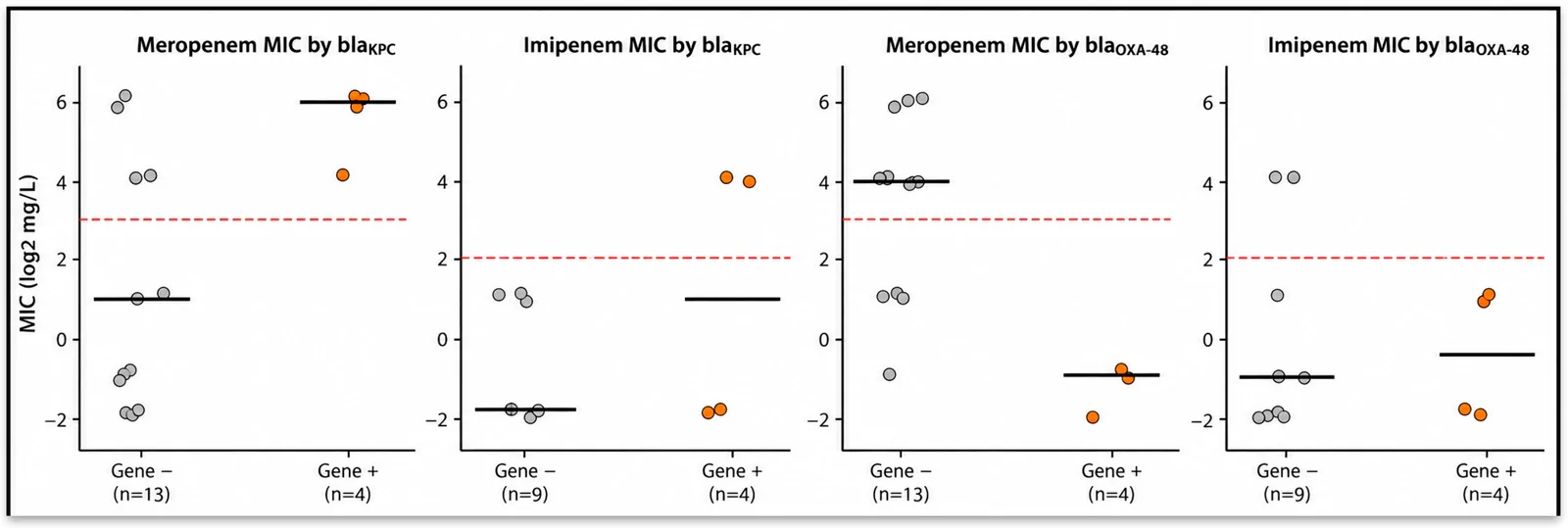
Carbapenem MIC distributions according to *bla*KPC and *bla*OXA-48 status among Enterobacterales with paired molecular and phenotypic results. Individual points represent isolates, and horizontal black lines indicate median MIC values. MIC values are plotted on a log_2_ scale. Dashed red lines indicate the EUCAST v15.0 resistance breakpoints for meropenem and imipenem. Gene-positive isolates are shown in orange and gene-negative isolates in grey. These MIC comparisons should be considered exploratory and interpreted cautiously because of the limited sample sizes.

The detailed concordance analysis for Enterobacterales is presented in Table 3. *bla*KPC demonstrated a sensitivity of 100% for imipenem resistance: all phenotypically imipenem-resistant Enterobacterales harboured the gene. However, 4 additional *bla*KPC-positive isolates were phenotypically susceptible, yielding a PPV of 0.50 (κ = 0.58; see Table 3 for exact 95% CIs). For meropenem, *bla*KPC showed lower sensitivity (0.44) but a PPV of 1.00 (κ = 0.43; Table 3 and Figure 3). MIC distribution analysis (Figure 4) confirmed that *bla*KPC-positive Enterobacterales showed substantially elevated meropenem MICs (median ≥16 mg/L) compared to gene-negative isolates (median ≤0.125 mg/L; *p* < 0.001).

In contrast, none of the four *bla*OXA-48-positive Enterobacterales included in each paired carbapenem analysis was phenotypically resistant to imipenem or meropenem. Consequently, sensitivity and PPV were 0 for both carbapenems. Specificity was 0.64 for imipenem and 0.50 for meropenem, with κ = −0.26 and −0.48, respectively (see Table 3 for exact 95% CIs). Given the small and imbalanced 2 × 2 tables underlying these estimates, these point estimates are reported descriptively rather than via qualitative agreement categories.

In *A. baumannii*, the three *bla*OXA-23-positive isolates were phenotypically imipenem-resistant, corresponding to 100% sensitivity and PPV within this small subset. Specificity and NPV could not be estimated because no gene-negative isolates were included.

*bla*KPC was detected in three *P. aeruginosa* isolates in the overall gene-distribution analysis. Because a complete isolate-level contingency table for KPC detection and carbapenem phenotype was not available for this species, formal measures of concordance were not calculated.

#### 2.2.2. β-Lactamase Genes (CTX-M, TEM)

*bla*CTX-M showed zero sensitivity for both ceftazidime (κ = 0.00) and cefotaxime (κ = 0.00) resistance. *bla*TEM was detected in only 2 isolates.

#### 2.2.3. Fluoroquinolone Resistance Markers

The *gyr*A83/87 mutation showed zero sensitivity for ciprofloxacin resistance (κ = 0.00), as did *qnr*A/B/S (κ = −0.20), that suggests other possible resistance mechanisms [17].

### 2.3. Carbapenem MIC Distributions According to Genotype

Carbapenem MIC distributions for Enterobacterales with paired molecular and phenotypic results are shown in Figure 4. The four *bla*KPC-positive isolates included in the meropenem analysis all had MIC values above the EUCAST resistance breakpoint, whereas the *bla*KPC-negative group contained both resistant and susceptible isolates. In the imipenem analysis, two of the four *bla*KPC-positive isolates were resistant and two were susceptible, consistent with the sensitivity of 1.00 but PPV of 0.50 reported in Table 3.

By contrast, all four *bla*OXA-48-positive isolates included in each paired analysis had imipenem and meropenem MIC values below the corresponding resistance breakpoints. Nevertheless, the gene-negative groups included both susceptible and resistant isolates. These distributions illustrate the poor discriminatory performance of *bla*OXA-48 detection for MIC-defined carbapenem resistance.

## 3. Discussion

This study provides a systematic evaluation of phenotype–genotype concordance between Unyvero multiplex-PCR resistance markers and MIC values obtained using the phenotypic AST comparator method in Gram-negative clinical isolates from a Romanian tertiary care center. Our findings reveal a markedly heterogeneous concordance landscape with direct implications for how molecular results should be translated into antibiotic treatment decisions.

The 78.8% non-susceptibility rate among Gram-negatives aligns with broader trends showing Romania among European countries with the highest carbapenem resistance rates [18,19]. The finding that *Escherichia coli, Klebsiella pneumoniae* and *Enterobacter cloacae* were highly resistant to carbapenems underscores resistance severity even outside ICU settings. These rates support a low threshold for molecular testing, as the probability of encountering a resistant organism is substantial. The 100.0% and 75.0% non-susceptible rates in *Acinetobacter baumannii* and *Pseudomonas aeruginosa,* respectively, confirm the multi-resistant nature of these nosocomial pathogens.

For *bla*KPC in Enterobacterales, sensitivity for imipenem resistance was high (2/2; 95% CI 0.16–1.00) and all *bla*KPC-negative isolates were imipenem-susceptible (NPV 9/9, 1.00; 95% CI 0.66–1.00), suggesting a potential role for *bla*KPC detection in the early identification of carbapenem-resistant Enterobacterales. However, PPV was limited (2/4, 0.50; 95% CI 0.07–0.93), so a positive result should be interpreted together with MIC-based susceptibility testing, particularly given the small number of isolates. Clinically, *bla*KPC detection may have potential implications for the early review of empirical therapy, since current guidance recommends agents such as ceftazidime–avibactam or meropenem–vaborbactam for infections caused by KPC-producing Enterobacterales [20], and meropenem–vaborbactam has also been evaluated in a randomized trial in this setting [21]. However, this study did not evaluate treatment decisions or patient outcomes, and any therapeutic decision should integrate the site and severity of infection, the patient’s condition, MIC-based susceptibility results, local guidelines and infectious-disease consultation.

The moderate PPV (0.50) warrants nuance: four *bla*KPC-positive isolates remained phenotypically susceptible. Gene detection establishes the presence of a determinant but not its expression, functionality, or contribution to the observed phenotype. Low-level expression, heteroresistance and sequence variation within *bla*KPC are recognised explanations for this pattern, but none was investigated here; resolving it would require expression analysis, sequencing of the detected alleles, and population-analysis profiling. Wang et al. [22] observed that <3.5% of KPC-producing Chinese CRE strains fell within the susceptible MIC range. A notable discrepancy was observed between imipenem and meropenem performance; the gene showed a sensitivity of 1.00 for imipenem, identifying all phenotypically resistant isolates, yet yielded a low PPV of 0.50. Conversely, for meropenem, *bla*KPC achieved a PPV of 1.00 but a diminished sensitivity (0.44), suggesting that while its presence consistently conferred resistance, additional undetected mechanisms contributed to the majority of the meropenem-resistant phenotypes. KPC functions within the periplasmic space. In the case of meropenem, the interplay between the porin-mediated influx rate and the KPC hydrolytic efficiency may be weighted in favor of the bacterium. Conversely, for imipenem, the balance may tip toward the antibiotic if porin permeability is high, allowing the drug to saturate the enzyme before complete degradation occurs [23].

Perhaps the most clinically significant finding is the zero sensitivity and negative kappa of *bla*OXA-48 for carbapenem resistance. All OXA-48-positive Enterobacterales were phenotypically carbapenem-susceptible. Because this analysis included only four *bla*OXA-48-positive isolates, this finding should be considered exploratory and should not be interpreted as evidence that *bla*OXA-48 detection predicts carbapenem susceptibility. These class D β-lactamases hydrolyse carbapenems weakly and require co-occurring ESBLs (typically CTX-M-15) or porin loss (OmpK35/OmpK36 deficiency) to confer clinically relevant resistance [8,9]. Abdelaziz [24] similarly reported significantly lower meropenem MICs for isolates carrying class D carbapenemases compared to class A + B genotypes. Poirel et al. [9] described OXA-48-like producers as “phantom menaces” because they may evade phenotypic detection while retaining the capacity for horizontal gene transfer.

Therefore a positive *bla*OXA-48 result cannot be taken as reassurance: in a critically ill patient, carbapenem-based therapy may be inappropriate even when in vitro carbapenem susceptibility is reported, and a fully active agent within a carbapenem-sparing regimen may be preferable [12,20,21]. A positive *blaOXA-48* result should therefore trigger confirmatory phenotypic testing (e.g., the carbapenem inactivation method), infection-control measures and cautious, susceptibility-guided therapy [25]. The present study did not evaluate treatment outcomes and therefore cannot determine whether carbapenems should be used or avoided in *bla*OXA-48-positive infections.

The absence of metallo-β-lactamase genes (*bla*NDM, *bla*VIM, *bla*IMP) in our cohort is noteworthy given recent Romanian data reporting that more than half of carbapenem-resistant *K. pneumoniae* co-produce NDM and OXA-48-like enzymes [18,19,26,27]. Several factors may explain this discrepancy: our cohort was drawn from a broad specimen mix (wound, respiratory, ear/sinus and blood) rather than being enriched for invasive *K. pneumoniae* bloodstream isolates; it derives from a single centre over a single year; and the local clonal background may differ from the referral-hospital series in which NDM co-production predominates. Because the panel does include MBL targets, their absence here represents a genuine local finding rather than a detection gap, but given the regional epidemiology it should be confirmed prospectively and cannot be generalized.

In *Acinetobacter baumannii*, *bla*OXA-23 showed concordance with phenotypic carbapenem resistance, consistent with the established role of OXA-23 as the predominant carbapenemase in this species worldwide [10]. But in our study, only three isolates were tested for *bla*OXA-23; therefore, we can consider the study as exploratory. Upon *bla*OXA-23 detection, clinicians should presume carbapenem resistance and consider colistin-based or high-dose sulbactam-based regimens. The *bla*KPC detection in only three carbapenem-resistant *Pseudomonas aeruginosa* isolates is noteworthy, as KPC-producing *Pseudomonas aeruginosa* represents an emerging threat in southern Europe [19]. However, the limited number of observations precludes definitive conclusions regarding concordance.

Zero sensitivity of *gyr*A 83/87 for ciprofloxacin is compatible with multi-mechanistic fluoroquinolone resistance involving *par*C mutations, efflux pumps, and plasmid-mediated quinolone resistance genes not targeted by the panel [28], although these were not tested experimentally. These mechanisms are plausible on the basis of the published literature but were not assessed in this study; whole-genome sequencing or targeted mechanistic testing would be required to establish them. These markers should be considered supplementary rather than definitive predictors.

*bla*NDM, *bla*VIM, or *bla*IMP are metallo-β-lactamases increasingly prevalent in Europe and the Eastern Mediterranean [24,25]. Abdelaziz [24] found *bla*NDM in 80.5% of Egyptian CRE, highlighting the significance of this gap. The Unyvero panel omits AmpC-type β-lactamases, porin gene mutations, and efflux pump regulators. A negative syndromic panel result should therefore never be interpreted as excluding resistance.

A further limitation intrinsic to syndromic panels is that, when run on polymicrobial specimens, a detected gene cannot be linked to a specific organism. Co-detection of a resistance gene and an organism should therefore not be interpreted as proof that the organism carries the gene unless supported by culture, AST and/or isolate-level molecular confirmation. In the present study, gene–species associations were restricted to monomicrobial cultures with matched identification, but this caveat is essential for the clinical use of such panels.

Acknowledging that this study did not assess clinical outcomes and was conducted at a single tertiary-care centre over a single year, our findings suggest the following potential implications for the interpretation of molecular results; these implications are specific to this cohort and cannot be generalized to other hospitals, regions, or patient populations without external, multicentre validation:

- *bla*KPC detected → suggests carbapenem resistance (high sensitivity, limited PPV in this cohort); the result may prompt early review of empirical therapy according to infection site, severity, local guidelines and infectious-disease advice, pending MIC-based AST; implement contact isolation.
- *blaOXA-48* detected → do not escalate empirical therapy; perform confirmatory phenotypic testing; implement infection control measures; monitor for emerging resistance.
- *bla*OXA-23 detected (*Acinetobacter baumannii*) → presume carbapenem resistance; consider colistin-based or sulbactam-based regimens.
- Negative panel → does not exclude resistance; maintain standard empirical therapy and await phenotypic AST results.

### Limitations

This study has several limitations. First, it was a single-centre, isolate-based study over one year, and several gene–drug analyses rested on very few gene-positive isolates, so all estimates are exploratory and imprecise. Second, MIC results came from an automated system used as the phenotypic comparator method, without broth microdilution confirmation, and we did not perform phenotypic confirmation of carbapenemase or ESBL production. Third, the panel detects β-lactamase genes at the gene level. It does not distinguish ESBL variants from non-ESBL variants of *bla*TEM and *bla*SHV, whose detection therefore does not imply an ESBL phenotype, and we did not interpret these genes as ESBL markers. Fourth, the panel does not target AmpC enzymes, porin alterations, efflux pumps or several fluoroquinolone resistance determinants, and we did not investigate these mechanisms. Finally, this study did not assess clinical outcomes, and its findings cannot be generalized to other hospitals, regions or patient populations without multicentre validation.

## 4. Materials and Methods

Study design and isolate collection. This cross-sectional observational study was conducted at the Department of Clinical Microbiology, Emergency County Hospital in Craiova, Romania, between January 2025 and December 2025. Consecutive, non-duplicate Gram-negative bacteria isolated from clinical specimens submitted for routine diagnostic purposes were eligible, irrespective of their carbapenem-susceptibility phenotype; both susceptible and non-susceptible isolates were retained so that concordance could be assessed across the full range of phenotypes. Isolates were obtained from clinical samples collected for routine diagnostic purposes from hospitalized adult patients. The study workflow is presented in Supplementary Table S2. To eliminate potential bias in susceptibility patterns, only the initial Gram-negative isolate per patient was included, regardless of the specimen source. Species identification was performed by using the VITEK^®^2 automated system (bioMérieux, Marcy-l’Étoile, France) with GN identification cards.

The 117 isolates included species such as *Klebsiella pneumoniae* (21), *Serratia marcescens* (15), *Escherichia coli* (12), *Enterobacter cloacae* (9), *Citrobacter freundii* (9), *Proteus mirabilis* (6), *Stenotrophomonas maltophilia* (18), *Acinetobacter baumannii* (15), and *Pseudomonas aeruginosa* (12). Because *Stenotrophomonas maltophilia* is intrinsically resistant to carbapenems—a property that would spuriously inflate genotype–phenotype discordance—it was excluded priori from the concordance analysis, leaving 99 isolates as the primary analysis population. For individual gene-antimicrobial comparisons, only isolates with both an available molecular result and an interpretable MIC result for the corresponding antimicrobial were included; therefore, the denominators varied between concordance analyses (Supplementary Table S1).

### 4.1. Phenotypic Antimicrobial Susceptibility Testing

MIC determination was performed using the VITEK^®^2 automated system (bioMérieux, Marcy-l’Étoile, France) with GN-series AST cards (N222, N427, N437, N438, GN75, XN05, XN26, Y233). Results were interpreted according to EUCAST clinical breakpoints v15.0 (2025) [29]. Quality control was performed using *Escherichia coli* ATCC 25922, *Pseudomonas aeruginosa* ATCC 27853, and *Klebsiella pneumoniae* ATCC 700603.

### 4.2. Multidrug Resistance Classification

An isolate was defined as non-susceptible when it was resistant to at least one tested antibiotic. Resistance profiles were classified according to the internationally harmonized definitions of Magiorakos et al. [30]: susceptible, single-drug resistant (SDR; non-susceptible to one antimicrobial class), multidrug-resistant (MDR; non-susceptible to ≥3 classes), extensively drug-resistant (XDR; susceptible to ≤1–2 classes). Because PDR requires demonstrated non-susceptibility to every category, including polymyxins and tigecycline, isolates for which these agents were not tested, or which remained colistin-susceptible, could not be classified as PDR.

### 4.3. Molecular Resistance Gene Detection

Resistance gene detection was performed using the Unyvero (Curetis GmbH/Bruker, Holzgerlingen, Germany) molecular multiplex-PCR analyser and HPN, BCU, and ITI cartridges, per manufacturer’s instructions. Markers evaluated: carbapenemases (*bla*KPC, *bla*OXA-48, *bla*OXA-23, *bla*NDM, *bla*VIM and *bla*IMP), β-lactamase genes (ESBL or non-ESBL variants not distinguished by the panel) (*bla*CTX-M, *bla*TEM, and *bla*SHV), aminoglycoside resistance (aacA4), fluoroquinolone resistance (*gyr*A 83/87, *qnr*A/B/S), and sulfonamide resistance (*sul*1). We used the cockpit software Curetis, version 6.0, assay version 30189rev. 2.0.

### 4.4. Statistical Analysis

Descriptive statistics were used to characterize the distribution of bacterial species and the prevalence of various resistance categories (SDR, MDR, XDR, and PDR). We excluded *S. maltophilia* isolates from statistical analysis because the intrinsic carbapenem resistance. Categorical variables, such as the proportions of multidrug-resistant (MDR) isolates between Enterobacterales and other Gram-negative bacteria, were compared using the chi-square test or Fisher’s exact test as appropriate.

Gene–antimicrobial pairs for concordance analysis were selected to represent the antimicrobial class each resistance determinant is expected to affect—imipenem and meropenem for the carbapenemase genes *bla*KPC, *bla*OXA-48, and *bla*OXA-23; ceftazidime and cefotaxime for the extended-spectrum β-lactamase gene *bla*CTX-M; and ciprofloxacin for the fluoroquinolone-resistance determinants *gyr*A83/87 and *qnr*A/B/S—rather than tested exhaustively against every antimicrobial agent on the susceptibility panel.

For each gene–antibiotic pair, a 2 × 2 contingency table was constructed using the Unyvero result as the index test and VITEK^®^2 MIC-based resistance classification as the phenotypic AST comparator method. Sensitivity, specificity, PPV, NPV, and Cohen’s kappa (κ) were calculated. Analyses were stratified by organism group: Enterobacterales, *Pseudomonas aeruginosa*, and *Acinetobacter baumannii*. Kappa interpretation followed Landis and Koch [31]: &lt;0 = less than chance; 0–0.20 = slight; 0.21–0.40 = fair; 0.41–0.60 = moderate; 0.61–0.80 = substantial; 0.81–1.00 = very good.

MIC distributions were compared between gene-positive and gene-negative isolates using box-and-whisker plots on a log_2_ scale and the Mann–Whitney U test. No multiplicity adjustment was applied. All analysis with a small number of isolates were declared as exploratory. All statistical tests were two-sided, and a *p*-value < 0.05 was considered statistically significant. Analyses were performed using STATA version 17.1 (StataCorp LLC, Lakeway Drive, TX, USA).

## 5. Conclusions

This study demonstrates that translating molecular resistance gene detection into antibiotic treatment decisions requires a gene-specific, context-dependent approach. *bla*KPC detection was associated with carbapenem resistance in Enterobacterales and may support early clinical decision-making, but should be interpreted together with MIC-based susceptibility testing. Conversely, *bla*OXA-48 detection does not correlate with phenotypic carbapenem resistance and should trigger confirmatory testing and infection control measures rather than automatic treatment modification. *bla*OXA-23 in *Acinetobacter baumannii* maintains high concordance and should prompt appropriate anti-*Acinetobacter* therapy. ESBL and fluoroquinolone markers showed poor standalone sensitivity, limiting their clinical utility as sole decision-making tools. These findings advocate for an integrated diagnostic strategy combining the speed of syndromic molecular panels with the accuracy of phenotypic MIC determination, contextualized within local resistance epidemiology, in order to optimize antibiotic stewardship for CPE and Gram-negative infections. Ultimately, antimicrobial treatment decisions should be based on the complete susceptibility profile, together with infection site, disease severity, pharmacological considerations, and the individual clinical context.

## Figures and Tables

**Table 1:** Distribution of Gram-negative isolates by species and multidrug resistance status (n = 99).

Non-Susc % [95% CI]	Non-Susc	XDR	MDR	SDR	Susc	N	Gram-Negative Species
100.0 [83.9–100.0]	21	9	9	3	0	21	*Klebsiella pneumoniae*
40.0 [16.3–67.7]	6	3	3	0	9	15	*Serratia marcescens*
100.0 [73.5–100.0]	12	5	7	0	0	12	*Escherichia coli*
100.0 [66.4–100.0]	9	3	6	0	0	9	*Enterobacter cloacae*
66.7 [29.9–92.5]	6	0	6	0	3	9	*Citrobacter freundii*
0.0 [0.0–45.9]	0	0	0	0	6	6	*Proteus mirabilis*
100.0 [78.2–100.0]	15	11	0	4	0	15	*Acinetobacter baumannii*
75.0 [42.8–94.5]	9	3	3	3	3	12	*Pseudomonas aeruginosa*
78.8 [69.4–86.4]	78	34	34	10	21	99	Total

Susc, susceptible; non-susc, non-susceptible to at least 1 antibiotic; SDR, single-drug resistant; MDR, multidrug-resistant; XDR, extensively drug-resistant; N, number; CI, confidence interval.

**Table 2:** Distribution of β-lactamase and carbapenemase genes across Gram-negative species.

Total	*bla*KPC	*bla*OXA-23	*bla*OXA-48	*bla*CTX-M	*bla*SHV	*bla*TEM	N	Species
12	3	0	3	3	0	3	21	*Klebsiella pneumoniae*
9	3	0	3	3	0	0	12	*Escherichia coli*
3	0	0	0	0	0	3	9	*Enterobacter cloacae*
3	0	0	3	0	0	0	9	*Citrobacter freundii*
6	0	0	3	3	0	0	15	*Serratia marcescens*
0	0	0	0	0	0	0	6	*Proteus mirabilis*
3	3	0	0	0	0	0	12	*Pseudomonas aeruginosa*
3	0	3	0	0	0	0	15	*Acinetobacter baumannii*
0	0	0	0	0	0	0	18	*Stenotrophomonas maltophilia*
39	9	3	12	9	0	6	117	Total

Total reflects cumulative gene detections per species. One isolate may carry multiple genes.

**Table 3:** Detailed diagnostic performance and agreement (κ) analysis for Enterobacterales resistance markers.

Marker	Agent	N	TP	FP	FN	TN	Sensitivity (95% CI)	Specificity (95% CI)	PPV (95% CI)	NPV (95% CI)	κ (95% CI)
Enterobacterales—primary carbapenemase analyses											
*bla*KPC	IPM	13	2	2	0	9	1.00 (0.16–1.00)	0.82 (0.48–0.98)	0.50 (0.07–0.93)	1.00 (0.66–1.00)	0.58 (−0.02 to 1.00)
*bla*KPC	MEM	17	4	0	5	8	0.44 (0.14–0.79)	1.00 (0.63–1.00)	1.00 (0.40–1.00)	0.62 (0.32–0.86)	0.43 (−0.05 to 0.91)
*bla*OXA-48	IPM	13	0	4	2	7	0.00 (0.00–0.84)	0.64 (0.31–0.89)	0.00 (0.00–0.60)	0.78 (0.40–0.97)	−0.26 (−0.99 to 0.48)
*bla*OXA-48	MEM	17	0	4	9	4	0.00 (0.00–0.34)	0.50 (0.16–0.84)	0.00 (0.00–0.60)	0.31 (0.09–0.61)	−0.48 (−1.00 to 0.60)
*Acinetobacter baumannii*											
*bla*OXA-23	IPM	3	3	0	0	0	1.00 (0.29–1.00)	NE—no gene-negative isolate	1.00 (0.29–1.00)	NE—no gene-negative isolate	NE
Enterobacterales—secondary, exploratory analyses											
*bla*CTX-M ^a^	CAZ	9	0	0	8	1	0.00 (0.00–0.37)	1.00 (0.02–1.00)	NE—no gene-positive isolate	0.11 (0.00–0.48)	NE ^a^
*bla*CTX-M ^a^	CTX	14	0	0	10	4	0.00 (0.00–0.31)	1.00 (0.40–1.00)	NE—no gene-positive isolate	0.29 (0.08–0.58)	NE ^a^
*gyr*A 83/87 ^a^	CIP	11	0	0	9	2	0.00 (0.00–0.34)	1.00 (0.16–1.00)	NE—no gene-positive isolate	0.18 (0.02–0.52)	NE ^a^
*qnr*A/B/S	CIP	11	0	1	9	1	0.00 (0.00–0.34)	0.50 (0.01–0.99)	0.00 (0.00–0.98)	0.10 (0.00–0.45)	−0.20 (−1.00 to 1.00)

TP, gene detected and phenotypically resistant; FP, gene detected and phenotypically susceptible; FN, gene not detected and phenotypically resistant; TN, gene not detected and phenotypically susceptible. The number following each estimate is its denominator: sensitivity is conditioned on resistant isolates, specificity on susceptible isolates, PPV on gene-positive isolates and NPV on gene-negative isolates. 95% confidence intervals are exact binomial (Clopper–Pearson); κ intervals use the Fleiss–Cohen–Everitt asymptotic standard error and are truncated at ±1. NE, not estimable. CAZ, ceftazidime; CIP, ciprofloxacin; CTX, cefotaxime; IPM, imipenem; MEM, meropenem; PPV, positive predictive value; NPV, negative predictive value. ^a^ No isolate in this analysis carried the marker, so sensitivity and PPV are uninformative and κ is degenerate (its confidence interval spans the entire range −1.00 to 1.00); these rows describe the absence of the marker among phenotypically resistant isolates and must not be read as evidence that the marker fails to predict resistance.

## Data Availability

The data supporting the findings of this study are not publicly available due to privacy and ethical restrictions.

## Supplementary Materials

The following supporting information can be downloaded at: https://www.mdpi.com/article/10.3390/antibiotics15101027/s1, Table S1. Isolate flow and analysis-set accounting. Table S2. Antimicrobial agents and reportable dilution ranges of the VITEK 2 AST-GN cards used for Enterobacterales susceptibility testing.
