# Supplementary material for: Concordance Between Multiplex-PCR Resistance-Gene Detection and MIC-Based Susceptibility in Gram-Negative Clinical Isolates from a Romanian Tertiary Hospital
Source: Antibiotics (Basel). 2026 Oct 15;15(10):1027. doi: 10.3390/antibiotics15101027 (PMC13700892; doi:10.3390/antibiotics15101027)
Supplement: Supplementary file 1 [file antibiotics-15-01027-s001.zip › antibiotics-4548885-supplementary.pdf]

Supplementary file

Supplementary Table S1. isolate flow and analysis-set accounting

Action	No. of isolates	Denominator
Consecutive Gram-negative isolates from clinical specimens, Jan–Dec 2025	118	–
Excluded: repeat isolate from a patient already represented	1	–
Underwent Unyvero testing	117	–
Unyvero run invalid / no result / repeated	5 (repeated)	–
Excluded from carbapenem concordance: *S. maltophilia* (intrinsic carbapenem resistance)	18	–
Primary analysis population	99	–
of which *Enterobacterales*	72	–
of which *P. aeruginosa*	12	–
of which *A. baumannii*	15	–
Paired analysis sets — isolates with both a valid marker call and an interpretable MIC		
bla$_{KPC}$ / imipenem (Enterobacterales)	13	59
bla$_{KPC}$ / meropenem (Enterobacterales)	17	55
bla$_{OXA-48}$ / imipenem (Enterobacterales)	13	59
bla$_{OXA-48}$ / meropenem (Enterobacterales)	17	55
bla$_{CTX-M}$ / ceftazidime (Enterobacterales)	9	63
bla$_{CTX-M}$ / cefotaxime (Enterobacterales)	14	58
gyrA 83/87 / ciprofloxacin (Enterobacterales)	11	61
*qnr*A/B/S / ciprofloxacin (Enterobacterales)	11	61
bla$_{OXA-23}$ / imipenem (*A. baumannii*)	3	12
bla$_{KPC}$ / carbapenems (*P. aeruginosa*)	0	12

There were 72 Enterobacterales (or 15 A. baumannii, 12 P. aeruginosa). The exclusion reason in the paired-analysis block is the relevant agent was not on the VITEK card used for those isolates.

Table S2. Antimicrobial agents and reportable dilution ranges of the VITEK 2 AST-GN cards used for Enterobacterales susceptibility testing

Cells give the lowest and highest reportable result (mg/L), where "≤" marks a left-censored and "≥" a right-censored value. "–" means the agent is not on the card. The second column gives the EUCAST v15.0 (valid from 2025-01-01) Enterobacterales MIC breakpoints, S≤/R> in mg/L. Agents are grouped by antimicrobial category (Magiorakos et al., 2012).

Agent (code)	EUCAST v15.0 S≤/R>	AST-N222	AST-GN75	AST-N233	AST-XN05	AST-N427	AST-N437	AST-N438	AST-XN26
Aminoglycosides									
Amikacin (AN)	(8)/(8)	≤2–≥64	≤2–≥64	≤2–≥64	–	≤1–≥64	≤1–≥64	≤1–≥64	–
Gentamicin (GM)	(2)/(2)	≤1–≥16	≤1–≥16	≤1–≥16	–	≤1–≥16	≤1–≥16	≤1–≥16	–
Tobramycin (TM)	(2)/(2)	≤1–≥16	≤1–≥16	≤1–≥16	–	≤1–≥16	–	–	≤1–≥16
Penicillins									
Ampicillin (AM)	8/8	–	≤2–≥32	≤2–≥32	–	–	≤2–≥32	–	≤2–≥32
Penicillins + β-lactamase inhibitors									
Amoxicillin/clavulanic acid (AMC)	8/8	–	–	≤2/1–≥32/16	–	–	≤4/2–≥64/2	≤4/2–≥64/2	–
Ampicillin/sulbactam (SAM)	8/8	–	≤2/1–≥32/16	–	≤2–≥32	–	–	–	≤2–≥32
Antipseudomonal penicillins ± β-lactamase inhibitors									
Piperacillin (PIP)	8/8	≤4–≥128	≤4–≥128	–	≤4–≥128	–	–	–	–
Piperacillin/tazobactam (TZP)	8/8	≤4/4–≥128/4	–	≤4/4–≥128/4	–	≤4/4–≥128/4	–	≤4/4–≥128/4	–
Ticarcillin (TIC)	NBP	≤8–≥128	–	≤8–≥128	–	–	–	–	–

Ticarcillin/clavulanic acid (TCC)	8/16	≤8/2–≥128/2	–	–	≤8/2–≥128/2	–	–	–	–
Temocillin (TEM)	0.001/16 (a, b)	–	–	–	–	–	–	–	≤4–≥32
Cephalosporins, 1st/2nd generation									
Cefazolin (CZ)	0.001/4 (a, b)	–	≤4–≥64	–	–	–	–	–	–
Cefalotin (CF)	NBP	–	–	≤2–≥64	–	–	–	–	–
Cephalexin (CN)	16/16 (a)	–	–	–	–	–	≤4–≥64	–	–
Cefuroxime (CXM)	0.001/8 (b)	–	–	–	≤1–≥64	–	–	–	≤1–≥64
Cephamycins									
Cefoxitin (FOX)	Screen	–	≤4–≥64	≤4–≥64	–	–	–	≤4–≥64	–
Cephalosporins, 3rd/4th generation									
Cefotaxime (CTX)	1/2	–	–	≤1–≥64	–	–	–	≤0.25–≥64	–
Ceftriaxone (CRO)	1/2	–	≤1–≥64	–	≤1–≥64	–	≤0.25–≥64	–	≤0.25–≥64
Ceftazidime (CAZ)	1/4	≤1–≥64	≤1–≥64	≤1–≥64	–	≤0.12–≥64	≤0.12–≥64	≤0.12–≥64	–
Cefepime (FEP)	1/4	≤1–≥64	≤1–≥64	–	≤1–≥64	≤0.12–≥32	–	≤0.12–≥32	–
Cefixime (CFM)	1/1 (a)	–	–	–	≤0.25–≥4	–	≤0.25–≥4	–	–
Cefpodoxime (CPD)	1/1 (a)	–	–	–	–	–	≤0.25–≥8	–	–
Monobactams									
Aztreonam (ATM)	1/4	≤1–≥64	–	–	≤1–≥64	≤1–≥64	–	–	≤1–≥64

Carbapenems									
Ertapenem (ETP)	0.5/0.5	–	≤0.5–≥8	≤0.5–≥8	–	–	≤0.12–≥8	≤0.12–≥8	–
Imipenem (IPM)	2/4	≤0.25–≥16	–	≤0.25–≥16	–	≤0.25–≥16	–	–	≤0.25–≥16
Meropenem (MEM)	2/8	≤0.25–≥16	≤0.25–≥16	–	≤0.25–≥16	≤0.25–≥16	≤0.25–≥16	≤0.25–≥16	–
Novel β-lactam/β-lactamase inhibitor combinations									
Ceftazidime/avibactam (CZA)	8/8	–	–	–	–	–	–	–	≤0.12–≥16
Ceftolozane/tazobactam (CT)	2/2	–	–	–	–	≤0.25–≥32	–	–	≤0.25–≥32
Imipenem/relebactam (IPR)	2/2 (b)	–	–	–	–	–	–	–	≤0.25/4–≥16/4
Meropenem/vaborbactam (MEV)	8/8	–	–	–	–	–	–	–	≤0.5–≥64
Fluoroquinolones and quinolones									
Ciprofloxacin (CIP)	0.25/0.5	≤0.25–≥4	≤0.25–≥4	≤0.25–≥4	–	≤0.06–≥4	≤0.06–≥4	≤0.06–≥4	–
Levofloxacin (LEV)	0.5/1	–	≤0.12–≥8	–	≤0.12–≥8	≤0.12–≥8	≤0.12–≥8	≤0.12–≥8	–
Moxifloxacin (MXF)	0.25/0.25	–	–	–	≤0.25–≥8	–	–	–	≤0.25–≥8
Ofloxacin (OFL)	0.25/0.5	–	–	≤0.25–≥8	–	–	–	–	–
Pefloxacin (PEF)	Screen	≤0.25–≥16	–	–	–	–	–	–	–
Nalidixic acid (NA)	Screen	–	–	≤2–≥32	–	–	–	–	–
Folate pathway inhibitors									
Trimethoprim/sulfamethoxazole (SXT)	2/4	≤20–≥320	≤20–≥320	≤20–≥320	–	≤20–≥320	≤20–≥320	≤20–≥320	–

Trimethoprim (TMP)	4/4 (a)	–	–	–	≤0.5–≥16	–	–	–	–
Glycylcyclines and tetracyclines									
Tigecycline (TGC)	0.5/0.5 (b)	–	–	–	≤0.5–≥8	≤0.5–≥8	–	–	≤0.5–≥8
Eravacycline (ERV)	0.5/0.5 (b)	–	–	–	–	–	–	–	≤0.12–≥8
Minocycline (MNO)	NBP	≤1–≥16	–	–	≤1–≥16	–	–	–	–
Tetracycline (TE)	NBP	–	–	–	≤1–≥16	–	–	–	–
Polymyxins									
Colistin (CS)	(2)/(2)	≤0.5–≥16	–	–	≤0.5–≥16	≤0.25–≥8	–	≤0.25–≥8	–
Phosphonic acids									
Fosfomycin (FOS)	8/8 (a, b)	–	–	–	–	–	≤16–≥256	≤16–≥256	–
Phenicols									
Chloramphenicol (C)	NBP	–	–	–	≤2–≥64	–	–	–	–
Other classes									
Nitrofurantoin (FT)	64/64 (a, b)	–	≤16–≥512	≤16–≥512	–	–	≤16–≥512	–	–
Rifampicin (RA)	NBP	≤2–≥32	–	–	–	–	–	–	–
Phenotypic test (qualitative)									
ESBL confirmation (FEP/CTX/CAZ ± clavulanate) (ESBL)	n/a	–	NEG/POS	–	NEG/POS	–	NEG/POS	–	NEG/POS
